# The relationship between psychological health and social support: Evidence from physicians in China

**DOI:** 10.1371/journal.pone.0228152

**Published:** 2020-01-29

**Authors:** Jiangjie Sun, Ruochuan Sun, Yuanyuan Jiang, Xiuyun Chen, Zhi Li, Zuqing Ma, Jiuchang Wei, Chengsen He, Liping Zhang

**Affiliations:** 1 Health Management College, Anhui Medical University, Hefei, Anhui, China; 2 Center for Data Science in Health, Anhui Medical University, Hefei, Anhui, China; 3 Department of General Surgery, The First Affiliated Hospital of Anhui Medical University, Hefei, Anhui, China; 4 Clinical Medical College, Anhui Medical University, Hefei, Anhui, China; 5 College of Nursing, Anhui Medical University, Hefei, Anhui, China; 6 School of Management, University of Science and Technology of China, Hefei, Anhui, China; 7 Center for Crisis Management Research (Sponsored by Beijing Planning Office of Philosophy & Social Science), School of Public Policy & Management, Tsinghua University, Beijing, PR China; 8 Department of Psychology, Anhui Medical University, Hefei, Anhui, China; University of Birmingham, UNITED KINGDOM

## Abstract

**Background:**

The psychological health (PH) of doctors affects the quality of medical service and is related to the safety of patients. The serious problems with the doctor-patient relationship in China can lead to long-term imbalances in doctor PH, and the poor PH status of doctors has raised scholars’ concern. Current research mainly focuses on how factors such as social support and the impact of the residential environment correlate with individual PH. We continue this direction of research to see how the mechanism of social support impacts physician PH, also investigating the moderating effect of demographic indicators on physician PH.

**Methods:**

Based on a survey of 399 physicians, a descriptive analysis of measured data was done using SPSS 19.0. Pearson correlation coefficient analysis was used to examine the correlations between PH and the social support rating scale (SSRS) and the demographic variables. KMO and Bartlett methods were used to examine the correlations between PH and SDS (a scale to measure depression) and between PH and SAS (a scale to measure anxiety). The method of factor analysis was used for multicollinearity tests, and multiple stepwise regression analysis was used to explore the demographic factors correlated with PH and SSRS. Two-way interactions in moderated multiple regression were used to test the moderating effect of education level and title on SSRS, SDS, and SAS.

**Results:**

Our results indicate that the level of PH is influenced by the age, education, and title of a doctor. A physician’s title is significantly and positively correlated with PH, but age and education are significantly negatively related. Age, education, and title also affect the level of SSRS in physicians. SSRS is positively correlated with age, education, and title, and SSRS positively influences PH. Education and title had significant effects on the moderating influences of SSRS, SDS, SAS, and PH.

**Conclusion:**

The factors directly affecting PH include SSRS, age, and title, while education was found to be an indirect influencing factor. To meet goals expressed in Chinese government policy related to these issues, we suggest strengthening the guidance of the media, introducing laws and regulations on doctor-patient risk management and control, reforming the review mechanism of hospital job titles, improving the education level of doctors, building a comprehensive evaluation system of “practice performance + doctor-patient satisfaction”, and strengthening doctor-patient empathy. Through such measures, the level of PH in physicians will improve.

## Introduction

In 1946, the third World Congress on Psychological Health (PH) defined psychological health as the development of an individual’s state of mind into an optimal state within the limits of maintaining physical, mental, and emotional compatibility with others [1]. A British study found that 10% to 20% of physicians suffer from depression at some point in their careers [2], and they have a higher risk of suicide than the general population. According to Connelly’s survey of Canadian physicians (2001), about 80% experienced emotional exhaustion [3]. Dowell (2000) found that the rate of psychological disorders among physicians in New Zealand was three times that of the general population [4]. Physicians are a special professional group, which is concerned with the quality of life and the physical and psychological health of the public. As the maintainers of others’ health, doctors’ own psychological health is particularly important and deserves social attention. However, a recent episode of the CCTV program “News 1+1” reported that the data of doctor-patient disputes showed a continuously rising trend. The doctor-patient conflict risk at hospitals has always existed but, especially in recent years, events of violence against medical professionals have occurred frequently [5], seriously affecting the PH of doctors.

### The relationship between anxiety, depression, and psychological health

Anxiety is an emotion caused by excessive worry about the safety of relatives or one’s own life, future, and destiny [6]. It includes worry, concern, sadness, tension, panic, and other components. Anxiety is associated with critical situations and unpredictable and unmanageable events. It is an emotional and behavioural response mode for human beings who are adapting and coping with their environment. If a person is anxious for a long time without objective reasons, afraid of catastrophe for no reason, or suffering from an incurable serious illness, the result will be restlessness, panic, and other symptoms. Such abnormal anxiety is a kind of mental illness.

Depression is usually caused by the sudden occurrence of major life events or by longstanding intense and unpleasant emotional experiences [7]. In the presence of increased negative emotions, a patient can become depressed and be constantly worried. Such people may estimate that their intelligence is too low and the difficulties around them are too great. Depression is an unhealthy psychological condition.

PH is mainly manifested in sound will, normal cognition, emotional coordination, a good adaptive state, and a complete personality. Zachary Walsh (2017) pointed out that depression and anxiety are the main manifestations of poor PH [8]. Anxiety can cause symptoms such as nervousness, restlessness, irritability, and sleep difficulties, and may also manifest itself as a component of withdrawal. It usually starts 1~3 days after the onset of anxiety and peaks within a week, lasting about 28 days [9–10].

Depression is often accompanied by slow thinking, reduced language communication, and decreased activity. It is hard to imagine how depression might affect a practising physician. Currently, research on PH from the perspective of anxiety and depression mainly focuses on related treatment studies, such as research on the treatment and application of anxiety [11–13]; analysis of the correlation between anxiety, cognition, and negative evaluation [14]; treatment of depression [15–16]); and correlations between depression, social stress, and other factors [17–20]. Recently, researchers showed evidence that anxiety and depression are related to the social environment and other factors [21–22]. Social support is one aspect of social environmental factors, especially in the medical and health industry. Social support may positively affect doctors’ confidence in diagnosis and treatment, enhance their sense of social responsibility, and improve their health.

### The status of physician PH and its relationship to social support

Social support means that individuals perceive or receive material support or emotional and spiritual support from individuals or groups of people [23]. Emotional and spiritual support includes empathy, attention, emotion, acceptance, trust, and encouragement. This support can provide a sense of social belonging, which can come through social networks and public opinions. Both social support and PH are important parts of social psychology. Thoits’ research (1983) on the influence of social support on PH caused by life stress events indicated that social support can enhance the protection of self-consciousness and reduce the individual’s psychological stress response [6], having a positive impact on the individual’s PH. Lau proposed that social support could alleviate the negative effects of stress events on people’s physical and psychological health, which supports Thoits’ opinion [7, 24–25], and psychological research also confirms this. Sarason et al. (1991) pointed out that cognition plays a key role in an individual’s perception of social support [26]; in other words, cognitive working model, plays an important role.

Individual cognition is also affected by demographic factors. Therefore, we propose that the influencing factors of social support on PH include gender, age, education, cultural background, individual personality, emotional expression, and other such factors. Depressed patients are more likely to show negative reactions [27]. The subjects of most psychological research efforts have been members of the general public, but doctors are a special occupation group. What is the effect of social support on the PH of doctors? What is the role of demographic factors in PH adjustment? In the current era of increasing doctor-patient risk, exploring the validity of the role of social support in alleviating the psychological problems caused by doctor-patient risk events is conducive to helping doctors to restore PH, improve doctor-patient communication, and improve the level of diagnosis and treatment, which has contemporary value and practical significance.

Based on the existing literature, we propose four hypotheses which relate to PH and SSRS, demographic characteristics, and departments. The following hypotheses are postulated.

Hypothesis 1: SSRS is positively correlated with PH.
Hypothesis 2: A significant difference in PH exists between different hospital departments.
Hypothesis 3: Demographic characteristics (age, gender, education, and title) significantly affect SSRS.
Hypothesis 4: Demographic characteristics (age, gender, education and title) are significantly related to PH.

**Fig 1:**
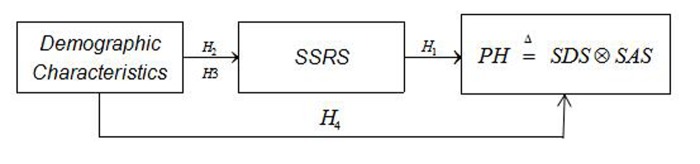
Conceptual model. Note: Each path is dentified by the hypothesis with which it is associated. SSRS: Social support rating scale, SDS:Self-Rating Depression Scale, SAS: Self-Rating Anxiety Scale, PH is redentified by SDS and SAS.

They form the conceptual model shown in Fig 1

## Methods

### Sample and data collection

The above hypotheses were tested using a survey of general hospital physicians in Anhui, China. The Yangtze River Delta urban agglomeration, constituted of Anhui, Jiangsu, Shanghai, and Zhejiang, is one of only six world-class urban agglomerations in the world. It has more than 60 million residents within its boundaries. Our research focuses on the landlocked central province of Anhui, whose political, economic, and cultural capital is the city of Hefei, in which most of this study’s physicians work. On June 14, 2019, the 17th China international talent exchange conference opened in Shenzhen, a conference at which the selections for “Charming China—the most attractive Chinese city in the eyes of foreign talents” was announced. Since 2018, Hefei has been ranked third in the list of “Chinese cities attracting talents”, with Shanghai ranked first and Beijing ranked second. Identified by the Economist Intelligence Units as one of the top 20 emerging powerhouses in China [28], Hefei had the fastest-growing metropolitan economy in the world in 2012 [29]. Tianchang, a model city of China’s medical reform, is located in the northeast of Anhui province and was another notable location of physicians studied in this survey.

**Fig 2:**
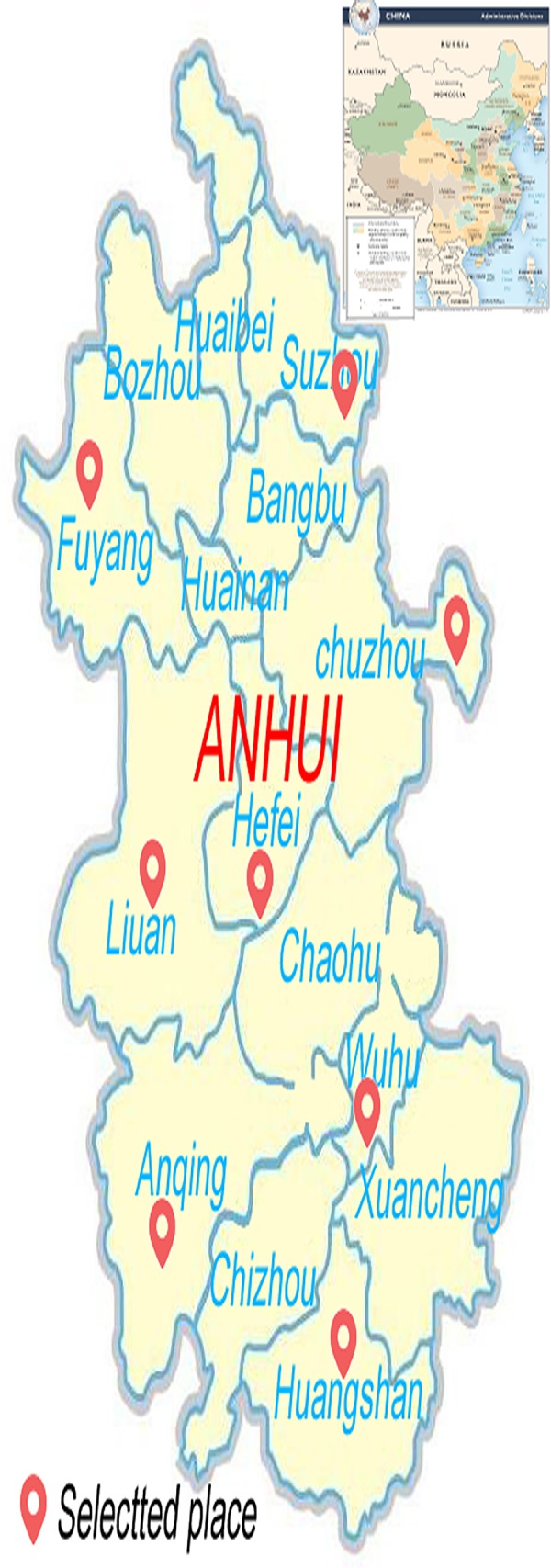
Location of Physicians studied in this PH research. The map of China in the picture is extracted from CIA website. Hint: the relative location of Anhui province, China and the world.

With the vigorous development of health care in Anhui Province, disputes between doctors and patients occur frequently, and the psychological sub-health state of doctors is very changeable. In this study, we selected doctors in comprehensive hospitals of Anhui province to explore the relationship between the PH of doctors and how much social support they receive. This study has guiding significance for improving tense doctor-patient relationships, and the results are representative of physicians across China. The specific research roadmap is shown in Fig 2

This study involves the whole of the province of Anhui, which is a trial area for China’s medical reform. Anhui is representative of many areas throughout China, and this reduces the bias of research results caused by regional economic differences, so it can be concluded that Anhui physicians in general hospitals are a suitable investigative sample for the study of SSRS and PH.

Ten comprehensive hospitals in Anhui province were selected for the study. The doctors surveyed included internists, surgeons, gynaecologists, paediatric specialists, and those from other clinical departments. The questionnaire was completed during the break of the hospital group meeting, and anonymous questionnaires were distributed to protect privacy of the study subjects. A total of 420 survey copies were issued, and 399 valid questionnaires were recovered, giving a response rate of 95%. Among the valid responses, 201 came from males and 198 from females. The average age of the subjects was 38.17 (SD = 8.718). For other demographic characteristics, see Table 1.

**Table 1:** Demographic characteristics of the subjects.

Variables		Number	Constituent ratio / %
Age / years			
	21~30	93	23.31
	31~40	145	36.34
	41~50	124	31.08
	>51	37	9.27
Education			
	Junior college	54	13.53
	College graduate	174	43.61
	Graduate/professional school	171	42.86
Title			
	Primary	57	14.29
	Intermediate	195	48.87
	Sub-senior	129	32.33
	Senior	18	4.51
HD			
	Internists	90	22.56
	Surgeon	101	25.31
	Gynecologist	64	16.04
	Paediatrics	79	19.80
	Other clinical departments	65	16.29

Notes: HD: Hospital department.

The survey was administered by researchers assisted by undergraduate students majoring in clinical medicine and health management, master of psychology students, psychology lecturers, and associate professors. This research team was trained before distributing the questionnaires. The questionnaire survey was conducted under the condition of informed consent of the respondents.

Considering the heavy workload of doctors and the large number of participants, the authors of the study signed a document committing themselves to obtain the verbal informed consent of all survey respondents. This approval procedure was approved by the Ethics Committee of Anhui Medical University.

### Measures used

#### Self-Rating Depression Scale (SDS)

Depression is a common negative emotion among individuals experiencing a bad family atmosphere, living pressure, career resistance, disharmonious working environments, and impetuous social environments. The occupation of physician has the characteristics of high work intensity, heavy tasks, and great responsibility, and the degree of depression in physicians is generally high. The issue of depression among physicians has drawn much attention from society [30]. Zung proposed the self-rating depression scale and verified its reliability and validity [31–32]. Subsequently, the scale has been widely used to measure group PH. For example, Kazama studied the relationship between mood disorders and mortality of dialysis patients in Japan [33], and the relationship between depression and living environment, and proposed the term “suburban neurosis” [34]. Also, there are other research results showing that the impact of living environment on individual depression is not obvious [35], and Rutter’s(2018) results showed that the risk of depression was related to the environment [22]. What is the depression status of doctors in China? Is there a relationship between physician depression and doctor-patient relationship? Does physician acceptance of social support reduce physician depression risk? These issues are worthy of attention.

The SDS was developed by Zung in 1965 [31]. In the current study, SDS was used to measure the level of psychological depression in the participants (Cronbach α coefficient 0.92, P<0.001). The SDS has 20 items in total, which are divided into four dimensions: mental-emotional (two items about depressed mood and crying), physical (8 items about daytime mood difference, sleep disturbance, decreased appetite, decreased libido, decreased body mass, constipation, tachycardia, and fatigue), psychomotor disorder (2 items: psychomotor hysteresis and agitation), and the psychological disorder (8 items about confusion, hopelessness, irritability, indecision, self-depreciation, emptiness, rumination about suicide, and dissatisfaction). For each item the response choices are “A little of the time” (1), “Some of the time” (2), “Good part of the time” (3), and “Most of the time” (4), and items 2, 5, 6, 11, 12, 14, 16, 17, 18, and 20 are scored inversely. The sum of the scores of all 20 items is the raw score, which is then multiplied by 1.25 and rounded to the nearest whole number to get the standard score.

The cutoff score for depression is 50. A score below 50 indicates no depression. A score greater than 50 needs to be noted, and the higher the score, the more the person will be prone to depression. People scoring more than 60 points should visit a psychologist for treatment.

#### Self-Rating Anxiety Scale (SAS)

From the form of scale construction to the specific evaluation method, the SAS is very similar to the SDS and is a clinical tool with strong operability for analysing patients’ subjective symptoms. It applies to adults with anxiety symptoms and complements the SDS in the diagnosis of psychological sub-health in patients, giving it extensive applications. International studies suggest that SAS can accurately reflect the subjective feelings of psychiatric patients with tendencies towards anxiety. The SAS is one of the common self-assessment tools for psychological consultation clinics, psychologists, and psychiatrists to understand anxiety symptoms. Zung proposed the SAS and verified its reliability and validity [36–37]. Subsequently, the scale has been widely used to measure group PH. For example, the SAS score of psychological patients is higher than that for people not receiving psychological treatment [36]. The scale has been widely used in the treatment of anxiety and depression [38], and in recognising the relationship between anxiety and mortality in dialysis patients in Japan [33]. In the situation of tense doctor-patient relationships in China, the anxiety level of doctors is generally high, and the excessive anxiety of doctors is of great concern to society [30].

Zung discovered the relationship between anxiety and living environment [34]. Rutter’s research showed that the risk of individual anxiety is related to the environment [22]. However, some research has shown that the impact of the living environment on individual anxiety is not obvious [35]. What is the anxiety status of doctors? What is the relationship between physician anxiety and the doctor-patient relationship? Does physician acceptance of social support reduce physician anxiety risk? These questions are all worthy of study.

In the current study, the SAS was used to measure the subjective feelings of the participants (Cronbach α coefficient 0.897, P<0.001), using a total of 20 items, including items, fear, panic, a sense of madness, a foreboding of misfortune, shaking of hands and feet, body pain, fatigue, inability to sit still, palpitation, dizziness, syncope, easy breathing, tingling of hands and feet, stomachache or dyspepsia, frequency of urinary motility, hyperhidrosis, facial flushing, sleep disorder, and nightmares.

The SAS mainly assesses the frequency of symptoms. It asks respondents to report their frequency level of symptoms of anxiety using the following scale: 1 –never or very seldom; 2 –sometimes; 3 –most of the time; 4 –most or all of the time. For example, 15 of the items are: “I feel more nervous and anxious than usual”, “I feel scared for no reason”,”I get upset or scared easily”, “I think I might go crazy”, “My hands and feet are shaking”, “I suffer from headaches, neck pain, back pain”, “I feel weak and tired easily”, “I feel my heart beat fast”, “I suffer from bouts of dizziness”, “I have fainting attacks or feel like I’m going to faint”, “My hands and feet are numb and tingling”, “I suffer from stomachache and indigestion”, “I often have to urinate”, “I blush and get hot”, and “I have nightmares”. The remaining five entries use the same scale but are scored inversely.

The scores of the 20 items were added as raw scores, and the totals were multiplied by 1.25, rounded to a whole number to get the standard score. According to the results of Chinese norms, the cutoff value of the SAS standard score is 50 points, with 50–59 classified as mild anxiety, 60–69 as moderate anxiety, and 70 or above as severe anxiety. In cases of moderate anxiety or worse, patients should visit a psychologist promptly for treatment. The higher the score, the higher the anxiety.

#### Psychological Health (PH)

Depression and anxiety are two basic characteristics of psychological representation, and they are also important indicators that reflect the PH of subjects [39]. In this study, the SDS and SAS were applied to explore the PH status of the subjects.

According to the sample data of physician mental health, combined with the action mechanism of each scale, we set SDS and SAS scores of less than 50 as indicators of physicians with healthy psychology. Scores between 50 and 60 for either SDS or SAS were taken to indicate sub-healthy psychology. If the SDS and SAS were both greater than 60, this was considered as indicative of unhealthy psychology in the physicians, requiring prompt medical treatment.

*Psychological state* is measured as unhealthy psychology = -1, sub-healthy psychology = 0, and healthy psychology = 1.

#### Social support rating scale (SSRS)

In the face of stress, difficulty, or stimulation, some people will suffer from depression, anxiety, and other emotions which are harmful to their health. Some people have a difficult time adapting, while others handle such situations well. The main influencing factors include social support, coping style, and individual psychological literacy differences [40–41]. Several recent studies have confirmed the association between social support and health status, such as the longitudinal association between social support and PH outcomes in patients with multiple sclerosis [42] and the association of different social support patterns with physical illness (hypertension, diabetes) or psychological illness [43]. Another example is a study of social support and PH status based on the older population in Tehran, Iran [44]. Further research showed that individual PH is positively correlated with social support, and good social support provides protection for individuals under stress [45]. However, is this true in China’s current tense doctor-patient relationships? If it is, how can the protective factor of social support be used to improve tense doctor-patient relationships? In this study, depression and anxiety are used as the characteristic variables of PH. In the current tense doctor-patient relationship environment in China, research on the correlation between PH and the social support of doctors certainly has innovative and timely value.

The SSRS was developed by Ganster in 1988 [46]. In the current study, the SSRS was used to measure the social support level of the participants (Cronbach α coefficient 0.80, P<0.001). The SSRS has 10 items in total, which are divided into three dimensions: objective support (three items about living conditions in the past year, problem-solving channels in emergency situations, and sources of psychological comfort in the event of stress or resistance); subjective support (four items about relationship with colleagues, relationship with neighbours, number of friends who can offer assistance, and level of support from family members); and support utilization (three items about the way one talks when in trouble, the way one asks for help when in trouble, and participation in group activities). Items are scored as follows. The respondent selects only one option for each item in items 1~5 and 8~9, scoring 1, 2, 3, and 4 points for item A, B, C, and D respectively. For items 6 and 7, the answer “no source” scores 0 points; otherwise, each source listed scores 1 point. Item 10 is divided into five subitems, and the total score is recorded. For each subitem, the response “none” scores 1 point, “rare” 2 points, “general” 3 points, and “full support” 4 points.

The total score is the sum of the scores of all ten items. The higher the score, the higher the social support level. A total score that is at most 22 is classified as a low level of social support, a score between 23 and 44 is classified as a medium level, and a score between 45 and 66 is classified as a high level.

#### Self-compiled questionnaire

The questionnaire included parts for gathering demographic data about the subjects (gender, age, education, title) and hospital department (internal medicine, surgery, gynaecology, paediatrics, and other departments). The data was formatted as follows: female = 1, male = 2. *Age* was measured in years. *Education* was a three-point variable with less than college = 1, college graduate = 2, and graduate/professional school = 3. *Title* has four categories, namely primary = 1, intermediate = 2, sub-senior = 3, and senior = 4. *Hospital department* has five categories, namely internal medicine = 1, surgery = 2, gynaecology = 3, paediatrics = 4, and other departments = 5.

#### Statistical methodology

Two graduate students reviewed all questionnaire data, and EpiData 3.1 was used for data entry. Using SPSS 19.0, a descriptive analysis was done for the measurement data (mean, standard deviation). Pearson correlation coefficient analysis was used to examine the correlation between PH (SDS and SAS) and SSRS and the demographic variables. KMO and Bartlett methods were used to examine the correlation between PH and SDS and between PH and SAS. The method of factor analysis was used for multicollinearity tests, and multiple stepwise regression analysis was used to explore the demographic factors of PH and SSRS. Two-way interactions in moderated multiple regression were used to test the moderating effect of education level and title on SSRS, SDS, and SAS. Differences are considered to be statistically significant when they reach the P<0.05 level.

## Results

According to the guidelines proposed by Comrey, a sample size of 100 is too small, whereas 200 is passable, 300 is excellent, 500 is good, and 1000 is very good [4,47]. For the general analysis of 40 item factors, in most cases, 200 samples are sufficient. Tinsley suggested that the subjects-to-items ratio be five to ten [48]. The sample size of our study was 399, so the subjects-to-items ratios for SDS, SAS, and SSRS were 19.95, 19.95, and 39.90 respectively, indicating that the amount of research data applied in the study met these criteria.

Based on the sample data of PH and SSRS, we created a pooled correlation matrix for each index and demographic data distribution (Table 2)

**Table 2:** Correlation matrix for the SSRS, SDS, SAS, PH, and demographic variables.

Variables	Mean	SD	1	2	3	4	5	6	7	8	9
1. Age	38.17	8.718	1								
2. Gender	1.50	0.501	-0.110	1							
3. Education	2.29	0.692	0.086	‐0.174	1						
4. Title	2.27	0.758	0.682	0.004	0.049	1					
5. HD	2.82	1.406	-0.002	-0.006	‐0.191	-0.022	1				
6. SAS	36.80	9.473	‐0.144	‐0.123	0.109	‐0.420	-0.050	1			
7. SDS	39.32	8.462	-0.031	‐0.124	0.218	‐0.332	-0.076	0.600	1		
8. PH	0.82	0.424	‐0.096	0.086	‐0.185	0.303	0.067	‐0.742	‐0.672	1	
9. SSRS	36.56	10.268	0.050	0.063	0.203	0.380	0.053	‐0.667	‐0.566	0.716	1

Notes: HD:Hospital department; PH:Psychological health. All significant at p < .05.

Table 2 gives descriptive statistics of physician PH, anxiety, depression, social support, and demographic variables, and also shows the correlations of variables. The correlations in the shaded part of Table 2 are statistically significant (P<0.05). SDS is positively correlated with SAS, PH is negatively correlated with SDS and SAS, and all r>.60 are significant at p < .05. All the significant correlations are highlighted. Therefore, it is feasible to use SDS and SAS as PH test indexes in this study. Generally, there will be a multicollinearity problem when the correlation coefficient is more than 0.9, and there may be a problem when the correlation coefficient is over 0.8 [49–50], so 0.6 is the baseline for an acceptable correlation coefficient [4]. Because SAS and SDS are two important influencing factors of PH, we used the KMO and Bartlett methods to test the correlation between PH and SAS and SDS (KMO = 0.71, P = 0.000). The results show that PH is significantly correlated with SAS and SDS. Pearson correlation analysis was performed for PH and SSRS (r = 0.72, P = 0.000); the results support Hypothesis 1, showing that social support positively affects physician PH.

For the demographic characteristic variables, only the correlation coefficient between Title and Age exceeds 0.6, at a value of 0.682. We conducted a multicollinearity test on all data. Often, the larger the variance inflation factor (VIF), the greater the problem of multicollinearity. More concretely, multicollinearity is not a problem if the tolerance value is greater than 0.10 or the variance inflation factors (VIFs) are less than 10 [4,51]. In our study, the lowest tolerance value is 0.532, and the highest VIF is 1.879. Accordingly, multicollinearity does not appear to be a significant problem in our dataset.

The regression model for PH and SSRS on demographic characteristic variables is provided in Table 3.

**Table 3:** Prediction of PH and SSRS.

	Step 1		Step 2	
Predictor	*β*/Coef	SE	*β*/Coef	SE
**PH**				
Age	-0.178***	0.003	-0.130***	0.002
Gender	-0.193	0.001		
Education	-0.023	0.001		
Title	0.077**	0.002	0.072***	0.002
HD	0.011	0.001		
SAS	-0.033***	0.002	-0.018***	0.002
SDS	-0.140**	0.002	-0.012***	0.002
SSRS	0.013*	0.002	0.012***	0.002
Adjust *R*^2^	0.680		0.716	
F	282.744***		201.556***	
**SSRS**				
Age	0.459***	0.071	0.436***	0.070
Gender	0.056	0.003		
Education	0.203***	0.004	3.009***	0.643
Title	0.875***	0.082	0.870***	0.080
HD	0.066	0.002		
Adjust *R*^2^	0.222		0.261	
F	57.743***		48.870***	

Notes

*, **, and *** indicate P<0.05, P<0.01, and P<0.001, respectively.

The data in Tables 2 and 3 do not support Hypothesis 2 but partially support Hypotheses 3 and 4. PH was significantly correlated with age and title, but not significantly correlated with gender, education, and HD. SSRS was significantly correlated with age, education, and title, but not significantly correlated with gender and HD. The results in Tables 2 and 3 also show that title is significantly correlated with age and education, and education is significantly correlated with gender and HD, respectively.

**Fig 3:**
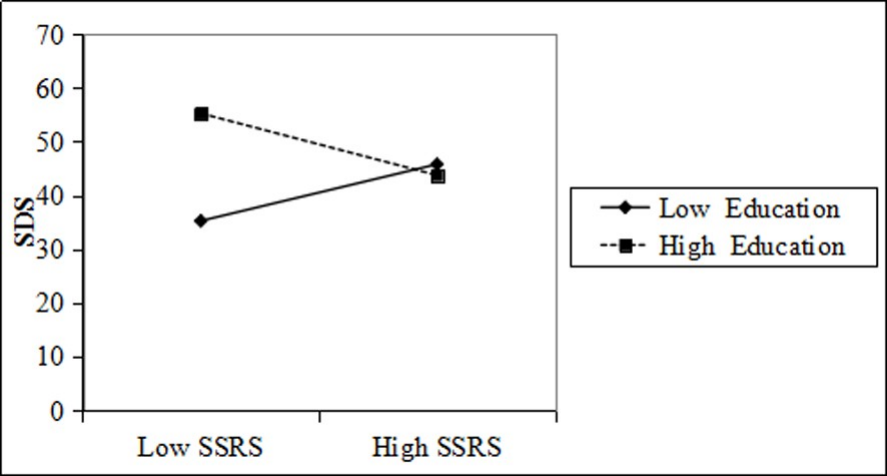
Education moderation interaction diagram for SSRS and physician SDS.

On this basis, we used two-way interactions in a moderated multiple regression to explore the moderating effect of education and title on SSRS, SDS, and SAS of physicians [52]. The specific results are shown in the following series of figures:

When physicians who were more highly educated received more social support, their depression scale scores were lower. When they received less social support, their scores on SDS were higher. In contrast, physicians who were less educated were less concerned about the social support they received (Fig 3).

**Fig 4:**
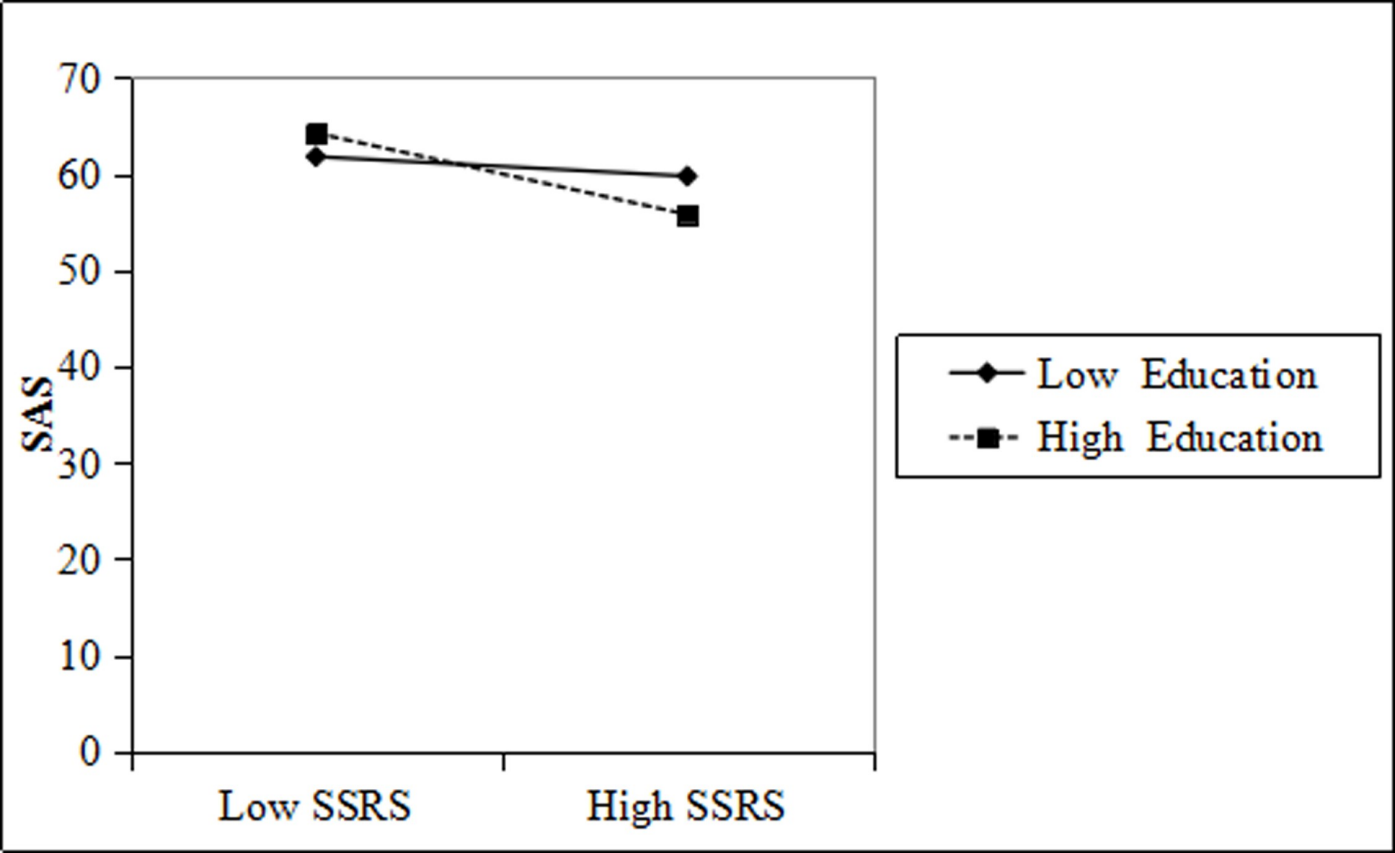
Education moderation interaction diagram for SSRS and physician SAS.

The psychological anxiety scores of doctors with high education and high social support were significantly lower than those with low education. The psychological anxiety scores of physicians with higher education and lower social support were significantly higher than those with lower education. More-educated physicians received more social support, and their depression scale scores were lower (Fig 4).

**Fig 5:**
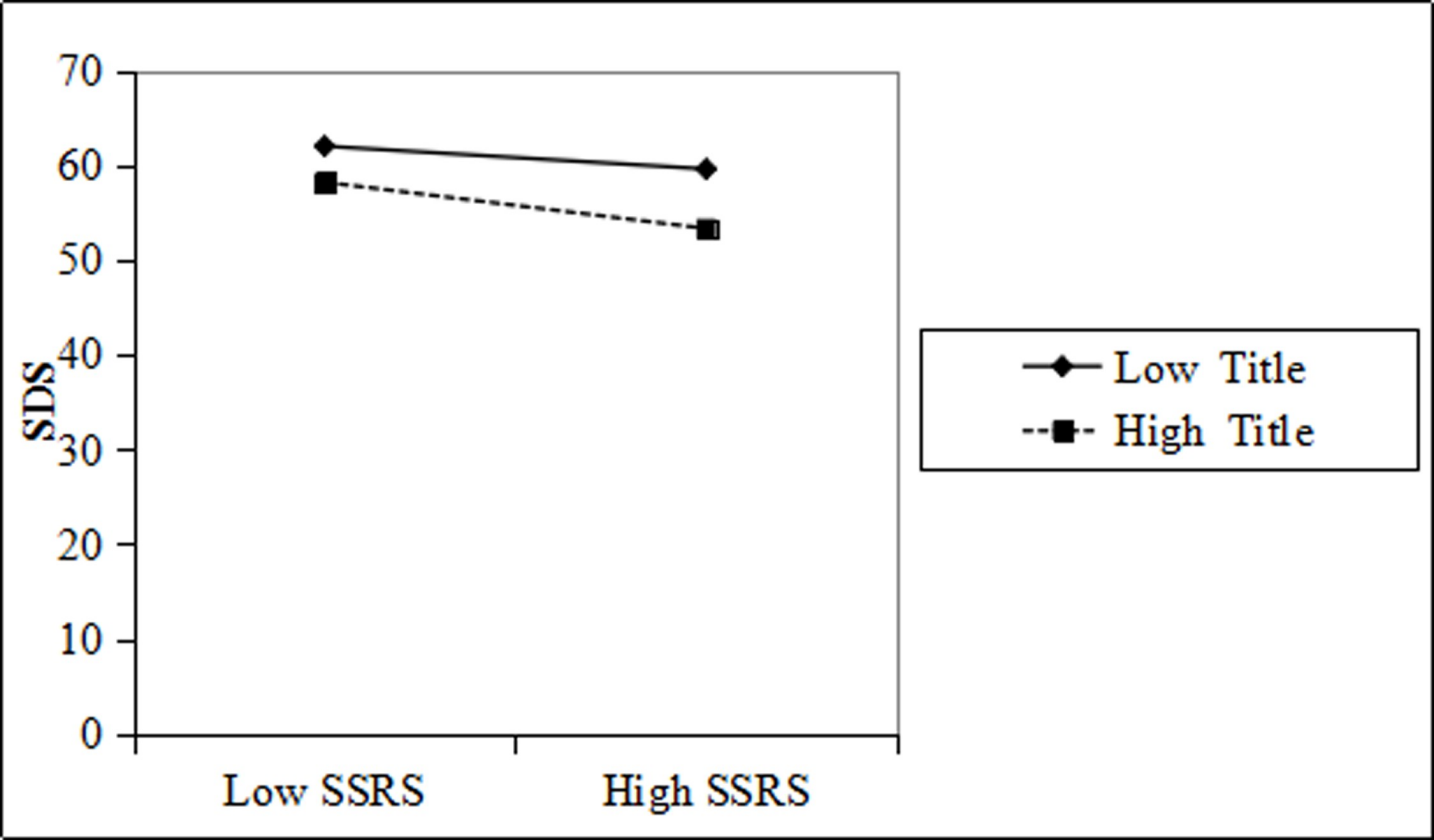
Title moderation interaction diagram for SSRS and physician SDS.

The SDS score of doctors with high titles was lower than that of doctors with low titles. With the same title, the higher the social support, the lower the SDS score (Fig 5).

**Fig 6:**
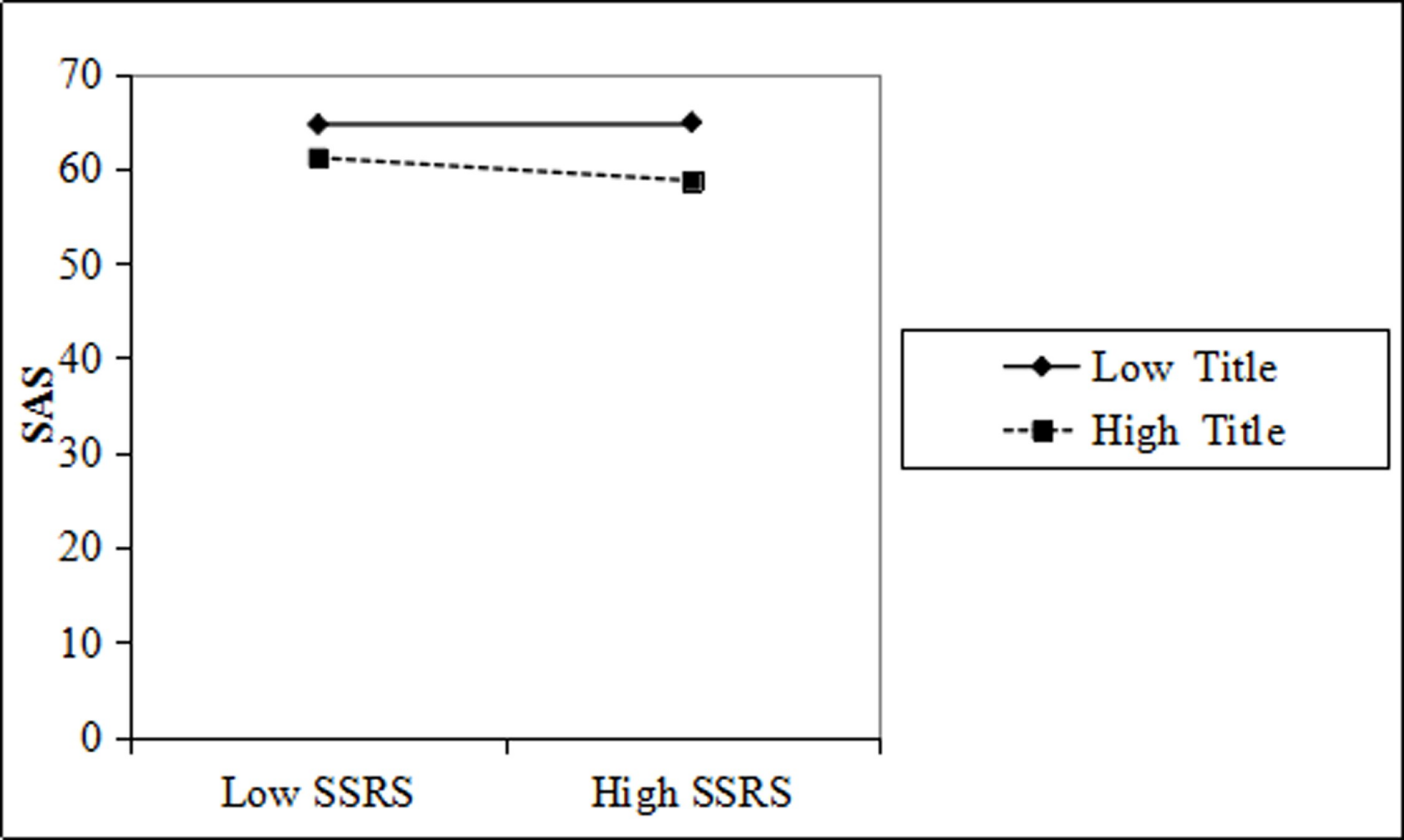
Title moderation interaction diagram for SSRS and physician SAS.

The SAS score of doctors with high titles was lower than that of doctors with low titles. With the same title, the higher the social support, the lower the SAS score, and the more obvious the effect of the higher title (Fig 6).

Figs 3, 4, 5 and 6 shows that education and title have a moderating effect on the SSRS, SDS, and SAS of physicians. Considering that the measurement indexes of PH are physician SDS and SAS, we further studied and obtained the regulatory validity of education and title on SSRS, as well as physician SDS, SAS, and PH. The relevant parameters are shown in Table 4.

**Table 4:** The moderating effect of physician education and title on SSRS, SDS, SAS, and PH.

Dependent	Independent	Moderator variable	Interaction coefficient	SE	R square change
SDS	SSRS	Education	-5.512 ***	1.026	0.138 ***
SAS	SSRS	Education	-1.605 ***	0.067	0.174 ***
SDS	SSRS	Title	-0.617 ***	0.022	0.122 ***
SAS	SSRS	Title	-0.661 ***	0.023	0.152 ***
PH	SSRS	Education	0.003 ***	0.001	0.009 ***
PH	SSRS	Title	0.012**	0.002	0.002**

Notes

** and *** indicate P<0.01 and P<0.001.

Table 4 further proves that education and title have significant regulatory effects on SSRS, SDS, SAS, and PH.

## Discussion

The results of this study show that the age, education, and title of doctors affected their PH. The PH of physicians was positively correlated with title and negatively correlated with age and education. One of the possible reasons is that doctors with high titles are more experienced in practising medicine, more skilled in analysing problems and dealing with the doctor-patient relationship, and more accepting of social support, so their mental health is better. These results are consistent with J. Sun’s research to some extent [39]. A second possible reason is that physicians with high education are mostly working in the front lines of medical care, with difficult tasks and heavy pressure. At the same time, although the education level of these physicians has generally improved, the competition for title promotion is intense, with frequent occurrences of uncivilised behaviour such as medical disputes, insult, physical hurt, and so on adding to the mental pressure. In particular, the older, highly educated physicians with lower titles often have more psychological pressure, a lack of self-confidence, low social support, psychological imbalances, negative emotions inducing mental sub-health, and even mental illness. This tendency is predictable and is also in line with an opinion expressed in previous research: “Frustration experience is an important cause of unhealthy psychology” [53].

In this study, the age, education, and title of doctors were positively correlated with their social support levels. A possible reason is that the older, more educated, and more professional physicians are, the higher their grasp of diseases, and the stronger their ability to solve patients’ problems, so it is understandable that they can win more social support. According to the data analysis shown in Table 2, it can also be concluded that the title of doctors is positively correlated with the age of doctors, which is in line with the career promotion mechanism of doctors in China and the Chinese hospital management system [4]. The negative correlation between the education level of doctors and gender is consistent with earlier research results [4]. A possible reason for the negative correlation between HD and education level is that in other studies, the factor “other departments” included hospital nursing, pharmacy, and laboratory services, and the education level of personnel in those three departments is lower than the average education level of hospital doctors.

The results of this study indicate that social support is an important protective factor of physicians’ PH. The more social support doctors receive, the better their PH status. Good social support can protect doctors involved in tense doctor-patient relationships. One reason is that “depression and anxiety”, the key factors that determine the PH of physicians, are mostly caused by the panic and uneasiness brought by the main environment and the peripheral environment in the cycle of states of emergency or stress. Objective support in physician social support includes support in living and working environments, among other factors. A good social doctor-patient relationship environment can give doctors a relaxed working environment, and the objective guidance of the media can make patients more grateful and doctors more dedicated. When grateful patients meet dedicated doctors, and empathetic doctors treat patients who ask for help, it is easy for doctors and patients to communicate with each other smoothly. Understandably, this reduces the risk of depression and anxiety in doctors and improves their PH. This is consistent with Rutter’s research entitled “The degree of fit of environmental dependence is related to depression and anxiety” [22].

Another possible reason that social support improves the doctors’ PH is that “depression and anxiety”, the key factors determining physicians’ PH, are mostly caused by poor mood caused by difficult family environments, living pressure, and disharmonic life and work environments. The subjective support of social support includes support from colleagues, neighbours, and family members. Under adverse circumstances, timely support from colleagues, neighbours, and family members can cultivate and improve physicians’ subjective feelings concerning such support and further enhance their subjective support. Only in this way can doctors’ symptoms of depression and anxiety be improved in the current tense doctor-patient relationship environment.

A third possible reason is that “depression and anxiety”, the key factors determining physicians’ PH, are mostly derived from physicians’ individual characteristics, such as their ability to adjust to adverse emotions under high stress. The degree of support utilisation in physician social support includes ways to relieve depression and anxiety. For example, older doctors with rich experience and high titles have relatively higher adaptability to stress than other physicians, so, understandably, they have better PH status as a whole. In conclusion, under high-stress conditions, the difficulty of coping that is caused by low social support or lack of social support has a serious, detrimental impact on PH.

## Conclusion

This study used Pearson correlation analysis to discuss the correlation between physician PH, social support, and demographic variables. Multiple stepwise regression analysis was used to explore demographic factors related to physicians’ PH and physicians’ social support, in order to explore how physician PH relates to social support and to verify the moderating effects of education level and title on the SDS, SAS, and PH.

The results of this study show that physician social support positively affected physician PH. Social support includes material assistance, behavioural assistance, information support, emotional support, and support from other resources. The more social support physicians receive, the richer their positive life experiences and positive emotional experiences, and the less the probability of negative emotions and a mental state that is not healthy.

From the theoretical perspective of psychology, the possible ways that physician social support affects the physician’s PH can be divided into two sources. First, social support has a buffering effect on physicians’ psychological stimulation and an indirect protective effect on physicians’ PH under high stress. This is consistent with the conclusion of Malinauskiene et al. [45]. Second, social support itself plays an independent role. Under the normal working conditions of doctors (not necessarily under high-stress conditions), good social support can maintain the positive emotional experience of doctors, thus benefitting their physical and psychological health. Therefore, we propose that the public understanding and support of the work of doctors; the scientific, objective, and correct leadership of social media; and the improvement of the current tension between doctors and patients are the fundamental countermeasures for solving the PH problems of doctors.

These results show that social support, age, and title were direct factors affecting the PH of doctors, and education level was an indirect factor affecting trust in doctors. Education and title have significant effects on the regulation of SDS, SAS, and PH. Accordingly, we propose firstly that laws and regulations, based on top-level design, be introduced to provide a good social environment for improving tense doctor-patient relationships. In this regard, Premier Li Keqiang of the Chinese State Council has signed Decree #701 of the State Council, and since October 1, 2018, regulations on the prevention and handling of medical disputes have been formally implemented. This policy embodies the concept of risk prevention and promotes research on doctor-patient risk prevention mechanisms to complement research on post-treatment of doctor-patient risk, laying a foundation for improving doctor-patient relationships. Secondly, it is necessary to promote the reform of the title evaluation system for doctors in China, make the professional positioning of doctors more scientific, and introduce into the title evaluation system comprehensive indicators of “practical performance + doctor-patient mutual evaluation”, so as to provide a driving force and form a mechanism for safeguarding the interests of doctors and patients. Thirdly, we further recommend that physician PH in China be promoted by the following measures: improving the education level of doctors, enhancing the confidence of doctors and patients’ trust in doctors, reducing the pressure of doctors’ work, enhancing the doctors’ professional sense of identity and social responsibility, and improving the patients’ empathy for the doctors’ profession.

The method used in this study was a questionnaire filled in by physicians, which entails some information bias. This study does not consider information such as the doctors’ marital status and the local GDP per capita, these factors are related to the doctors’ personal economic situations, can be used as indicators quality of their life, and associated with Doctor's PH. Although more factors should add to the findings. In fact, the focus of our study was that demographic factors ARE correlated to SSRS and PH, the authors believe it may not affect the results and findings of this study. Further research could include an expansion of the sample size, the use of comparison and control methods, and the provision of dynamic management information which might provide a better understanding of the PH predictions revealed in this paper.

## Supporting information

S1 Dataset Data of this study. (XLSX)
